# Radiological Emergencies and the Medical Physicist

**DOI:** 10.1120/jacmp.v13i1.3799

**Published:** 2012-01-05

The widespread damage and loss of life caused by the March 11th earthquake‐generated tsunami in Japan, and the resulting emergency at the Fukushima Daiichi nuclear power facility, raise once again the question of the role of knowledgeable civilians in responding to public health emergencies. In the case of a radiological emergency, medical physicists are among the more knowledgeable individuals in the private sector with regard to several relevant issues including radiation exposures and risks, radioactive contamination, and the fear of radiation that makes management of a radiological emergency so difficult. The challenges for medical physicists are to know the consequences of radiation exposure and radioactive contamination, to separate fantasy from fact in dealing with these consequences, and to address the consequences in a manner that instills confidence rather than fear in those who are less knowledgeable. All medical physicists should be knowledgeable about radiological emergencies and be prepared to respond to an emergency if one occurs in their vicinity.

Four types of radiological emergency would potentially expose large numbers of people to high amounts of radiation and require an emergency response that involves medical physicists. These events are: (1) the detonation of a nuclear weapon or improvised nuclear device, with an impact on the order of the Hiroshima and Nagasaki nuclear bombs, or possibly larger; (2) a crisis at a nuclear power plant, including a possible core meltdown and the release of radioactive contamination, such as is possible at the Fukushima Daiichi nuclear complex; (3) activation of an explosive radiological dispersal device, sometimes termed a “dirty bomb”; and (4) placement of a hidden radioactive source in a highly‐populated area where many people could be exposed to substantial doses of radiation. Somewhat less threatening but still of major concern is a transportation accident involving a cargo with high levels of radioactivity. One or more of these events is conceivable anywhere in the world. Should one occur, medical physicists in the vicinity will be recruited as experts in the management of the aftereffects of the event, including the possible exposure of many individuals to radiation and/or radioactive contamination. The question every medical physicist should ask is, “How prepared am I to respond if called upon to help in the management of a radiological emergency?”

There are several information sources that a medical physicist can access to expand his/her preparedness for a radiological emergency. A good place to start is a recent article published in *Radiology* entitled “Medical Response to a Radiological Emergency: A Primer for Medical and Public Health Practitioners”.^(^ ^1^ ^)^ This article has been posted for unrestricted access on *Radiology*'s home page.^(^ ^2^ ^)^ In addition to an extensive reference list, the article has a section entitled “Additional radiological emergency response information and resources”. This article is not sufficient to prepare a medical physicist completely to respond to a radiological emergency, but it is a good introduction or refresher to what the physicist needs to know.

Several other excellent sources of information exist, including publications of the International Atomic Energy Agency.^(^ ^3^ ^)^ Organizations such as the American Association of Physicists in Medicine may wish to offer educational sessions on preparing medical physicists to respond to radiological emergencies at their annual meetings.

In the United States, an excellent education and training resource for learning how to respond to public health emergencies, including radiological events, is the Medical Reserve Corps (MRC).^(^ ^4^ ^)^ The MRC is a volunteer organization that is sponsored by the Office of the US Surgeon General. Its mission is “to engage volunteers to strengthen public health, emergency response and community resiliency”. MRC regional units are community‐based, and provide a way to locally organize and use volunteers who donate their time and expertise to prepare for and respond to emergencies, including radiological events. MRC volunteers supplement existing emergency and public health resources, and include physicians, nurses, pharmacists, dentists, veterinarians, and epidemiologists, among others. A few medical physicists participate in the MRC, but many more are needed. Individuals participating in the MRC receive extensive training in emergency response and are credentialed to help mount an effective response to a public health emergency. The training includes not only on‐site immediate and triaging activities, but also efficient management of victims during transport and initial medical care. Volunteers participate in training exercises and response planning for public health emergencies of various types. Some other countries may have similar opportunities for volunteers concerned about public health emergencies.

Medical physicists are committed to maintaining the safety of patients in their facilities. But their responsibilities extend beyond their facilities and into their communities. For the latter, knowledge of the challenges of a radiological emergency — and how to respond to it — is an essential component of the medical physicist's knowledge and training

Dr. William R. Hendee

Editor‐in‐Chief of *Medical Physics*

## References

[1] Wolbarst A , Wiley A , Nemhauser J , Christensen D , Hendee W . Medical response to a major radiologic emergency: a primer for medical and public health practitioners. Radiology 2010;254(3):660–77. 20177084 10.1148/radiol.09090330

[2] http://radiology.rsna.org.

[3] www.iaea.org.

[4] www.Medicalreservecorps.gov.

